# Metabolomic machine learning-based model predicts efficacy of chemoimmunotherapy for advanced lung squamous cell carcinoma

**DOI:** 10.3389/fimmu.2025.1545976

**Published:** 2025-04-02

**Authors:** Liang Zheng, Wei Nie, Shuyuan Wang, Ling Yang, Fang Hu, Meili Ma, Lei Cheng, Jun Lu, Bo Zhang, Jianlin Xu, Ying Li, Yinchen Shen, Wei Zhang, Runbo Zhong, Tianqing Chu, Baohui Han, Xiaoxuan Zheng, Hua Zhong, Xueyan Zhang

**Affiliations:** ^1^ Department of Respiratory and Critical Care Medicine, Shanghai Chest Hospital, Shanghai Jiao Tong University School of Medicine, Shanghai, China; ^2^ Department of Ultrasonography, Shanghai Chest Hospital, Shanghai Jiao Tong University School of Medicine, Shanghai, China; ^3^ Department of Thoracic Medical Oncology, The Cancer Hospital of the University of Chinese Academy of Sciences (Zhejiang Cancer Hospital), Zhejiang, Hangzhou, China; ^4^ Hangzhou Institute of Medicine (HlM), Chinese Academy of Sciences, Zhejiang, Hangzhou, China; ^5^ Department of Respiratory Endoscopy, Shanghai Chest Hospital, Shanghai Jiao Tong University School of Medicine, Shanghai, China

**Keywords:** metabolomics, machine learning, chemoimmunotherapy, predictive model, tumor biomarkers

## Abstract

**Background:**

Unlike lung adenocarcinoma, patients with advanced squamous carcinoma exhibit a low proportion of driver gene positivity, with fewer effective treatment strategies available. Chemoimmunotherapy has now become the standard first-line treatment for individuals diagnosed with advanced lung squamous carcinoma. Serum metabolomics holds significant potential for application in predicting responses to chemoimmunotherapy and is capable of identifying and validating potential biomarkers. The aim of our study was to establish a model that can predict the prognosis of chemoimmunotherapy in patients with advanced lung squamous cell carcinoma, integrating metabolomics with machine learning techniques.

**Methods:**

We collected 79 serum samples from patients with advanced lung squamous cell carcinoma before receiving combined immunotherapy and performed untargeted metabolomics analysis. Patients were divided into non-response (NR) and response (R) groups according to overall survival (OS), and prognostic models were constructed and validated using different machine learning methods. The patients were further categorized into high-risk and low-risk groups based on the median risk score, to assess the model's predictive performance.

**Results:**

There were significant differences in metabolites and metabolic pathways between NR and R groups, and 117 differential metabolites were preliminarily screened (p < 0.05, VIP > 1). Further, least absolute shrinkage and selection operator (LASSO) and random forest (RF) were used to identify metabolites, and then their common metabolites were used as the best biomarkers to build a prediction model containing 8 differential metabolites. Based on these biomarkers, RF, support vector machine (SVM) and logistic regression were used to randomly divide patients into training and validation sets in a 7:3 ratio, respectively. We found that the RF method resulted in area under curves (AUCs) of 0.973 and 0.944 for the training and validation sets, respectively, with the best predictive performance. Subsequently, both OS and progression-free survival (PFS) were notably reduced in the high-risk group when contrasted with the low-risk group.

**Conclusions:**

We developed a model containing 8 metabolites based on metabolomics and machine learning that may predict survival outcomes in patients with advanced lung squamous cell carcinoma undergoing chemoimmunotherapy, helping to more accurately assess efficacy and prognosis in clinical practice.

## 1 Background

Lung cancer is one of the malignant tumors with the highest incidence rate and mortality in the world (1, 2). Non-small cell lung cancer (NSCLC) accounts for about 80%-85%, including histological subtypes such as adenocarcinoma and squamous cell carcinoma (3). Among them, squamous cell carcinoma accounts for about 30% of NSCLC and is a common type of lung cancer (4). Moreover, squamous cell carcinoma often remains asymptomatic in its initial stages. However, by the time it is detected, the disease may have advanced to a later stage, resulting in a less favorable prognosis (5). Compared to patients with lung adenocarcinoma, due to the limited number of driver gene mutations in patients with advanced lung squamous cell carcinoma, the options for targeted therapy are limited, and chemotherapy was once the main treatment method (6, 7). However, patients receiving chemotherapy alone often develop drug resistance quickly and have some adverse effects. With the development of immunotherapy, the treatment strategies for lung squamous cell carcinoma have undergone significant changes. Immunotherapy, which activates or enhances the patient's own immune system to attack tumor cells, has become an important component of the treatment of lung squamous cell carcinoma (8).

In recent years, multiple clinical studies have shown that immunotherapy combined with chemotherapy can significantly improve the survival of patients with lung squamous cell carcinoma, such as the CheckMate 017 (9) and CheckMate 078 studies (10). Immunotherapy biomarkers for lung squamous cell carcinoma mainly include PD-L1 (programmed cell death ligand 1) expression levels and tumor mutation burden (TMB). However, it has been shown that PFS was significantly superior in the combination arm regardless of PD-L1 expression level (11). This suggests that the efficacy of the combined regimen in patients with advanced squamous cell carcinoma cannot be predicted based on PD-L1 expression levels alone. TMB is also controversial as a biomarker to predict the efficacy of immunotherapy plus chemotherapy, especially there is no uniform standard for the selection of TMB detection methods and thresholds (12). Therefore, it is crucial to discover more new biomarkers to more accurately identify which advanced lung squamous cell carcinoma patients with negative driver genes are most likely to benefit from immune combination therapy.

Traditional biomarkers tend to be obtained from tumor specimens at a single time point, are invasive, cannot dynamically monitor changes in the tumor immune microenvironment, and there is heterogeneity in tumor tissues, which are limitations of past biomarkers. Blood specimens can be obtained more easily, causing less trauma and discomfort to the patient, and repeated and multiple sampling can be performed (13). It is also able to track the dynamic changes during treatment, reflect tumor and host microenvironment changes, and compensate for the limitations of biopsy or puncture samples that cannot obtain the full picture of the tumor (14). Metabolites in blood identify early biochemical changes in disease and have been widely used in disease prediction (15). With the development of omics technology, especially metabolomics technology, it has become a hot spot to use multiple metabolomics feature profiles and integrate multiple biomarkers based on Artificial Intelligence (AI) modeling to improve disease prediction accuracy (16). We found that previous studies have constructed early screening models for lung cancer based on metabolomics (17, 18), but there is still a lack of exploration in constructing models to predict the efficacy of immunotherapy combined with chemotherapy for advanced lung squamous cell carcinoma.

Therefore, our study aimed to compare metabolic pathways and metabolite differences between different efficacy in driver gene-negative advanced squamous cell carcinoma of the lung patients receiving first-line immunotherapy combined with chemotherapy using serum untargeted metabolomics. Prediction models were constructed based on various methods such as least absolute shrinkage and selection operator (LASSO), random forest (RF), support vector machine (SVM), and logistic regression (LR) to explore potential biomarkers that may predict survival outcomes in patients with advanced squamous cell carcinoma of the lung who are driver gene negative.

## 2 Materials and methods

### 2.1 Patients

We retrospectively screened 3124 patients treated at Shanghai Chest Hospital from July 2020 to July 2021, and finally included 79 patients who met the criteria and ended follow-up on April 31, 2024. All patients provided written informed consent, which was approved by the Ethics Committee and Institutional Review Board of Shanghai Chest Hospital (Reference number: LS1808). The inclusion criteria were: (1) histologically or cytologically confirmed squamous cell carcinoma of the lung; (2) stage IIIB to IV according to the ninth version of TNM, which was not suitable for radical surgery; (3) having measurable lesions; and (4) receiving first-line PD-1 (programmed cell death 1) inhibitor combination chemotherapy. (5) Eastern Cooperative Oncology Group (ECOG) performance status (PS) score between 0 and 1. Exclusion criteria were: (1) presence of driver gene mutations; (2) surgical treatment after immunotherapy; (3) active infection, such as HIV, hepatitis B, hepatitis C, etc.; (4) incomplete clinical data or did not complete the necessary systemic examination; (5) patients with other primary active malignancies; (6) patients with a history of autoimmune diseases, severe cardiopulmonary dysfunction or other serious complications.

We collected clinical information on patients' age, sex, smoking history, TNM stage, PD-L1 expression, ECOG PS, etc. via the hospital 's electronic system of medical records. Patients were treated with PD-1 inhibitors combined with chemotherapeutic agents administered every 3-4 weeks as a cycle until disease progression or serious adverse reactions or death. Immunologic medications included pembrolizumab, tislelizumab, cariselizumab, and sintilimab at a single dose of 200 mg. According to the patient 's body surface area and tolerance, the specific chemotherapy drug administration regimen was generally platinum-based doublet, platinum drugs included carboplatin, cisplatin, lobaplatin or nedaplatin, drugs used in combination with platinum drugs included paclitaxel drugs, gemcitabine or docetaxel; for patients not suitable for platinum drugs, gemcitabine combined with vinorelbine or gemcitabine combined with docetaxel is given. Patients' condition will be assessed before each cycle by chest computed tomography (CT), abdominal ultrasound, bone scan, brain enhanced magnetic resonance imaging (MRI) or positron emission tomography-computed tomography (PET-CT), and progression will be judged by at least one professional radiologist and clinical medicine.

### 2.2 Serum samples collection and pretreatments

Our serum samples were obtained with prior approval from the Shanghai Chest Hospital Clinical Biobank and could be investigated with these samples. Serum samples were previously stored in a -80°C freezer, and we obtained serum samples and performed untargeted metabolomic analysis within 4 weeks before patients received immune combination therapy by consulting patients' blood collection time records.

Serum samples stored in a -80°C freezer were preprocessed for analysis, first thawed on ice and vortexed, and 50 μL of sample was mixed with 300 μL of extraction solution (acetonitrile: methanol = 1:4, volume ratio) in a 2 mL microfuge tube. Vortex again, centrifuge, collect 200 μL of supernatant, place the collected supernatant at -20°C for 30 mins, then centrifuge to obtain 180 μL of supernatant for liquid chromatography-mass spectrometry (LC-MS) analysis (19).

### 2.3 Untargeted metabolomics study

Pretreated serum samples were analyzed by LC-MS in the next step, where the specific setting of the chromatographic part was the column Waters ACQUITY UPLC BEH C18, 1.8 µm particle size, 2.1 mm inner diameter x 100 mm length; the column temperature was 40°C, the flow rate was 0.4 mL/min, and the volume of sample injected into the column was 2 μL. Gradient elution was performed in a solvent system containing 0.1% formic acid in water (mobile phase A) and 0.1% formic acid in acetonitrile (mobile phase B) in steps. That is, 5% mobile phase B started at 0 min; within 11 min, a linear gradient to 90% mobile phase B; 90% mobile phase B was held for 1 min; return to 5% mobile phase B within 0.1 min and hold for 1.9 min to quickly return to starting conditions and prepare for the next injection.

The mass spectrometer was TripleTOF 6600, the data acquisition software was Analyst TF 1.7.1 (Sciex, Concord, ON, Canada), the mass spectrometry acquisition mode was information-dependent acquisition (IDA) mode, and appropriate parameters were set to ensure high sensitivity and resolution of LC-MS analysis. In addition, we prepared a pooled human serum sample from all study participants as a consistent quality control (QC) sample. Then, a blank solvent was used as a negative control. These QC samples were evenly distributed throughout the analysis batches. Specifically, they were inserted every 10 samples to monitor the stability and consistency of the analytical process. For the pooled human serum, we monitored the relative standard deviation (RSD) of the peak areas for major metabolites. An RSD threshold of 30% was set, and any metabolite exceeding this threshold was excluded from further analysis. For the blank solvent, we checked for any carryover effects by ensuring no significant peaks were observed that could indicate contamination from previous samples. It can be used to assess the quality of the data and also to monitor within-run and between-run variability during the analysis. Sample handling and repeatability of the analytical method can be assessed by comparing the results of QC samples analyzed at different time points.

Raw data files generated by the mass spectrometer such as mzML or raw format were converted to mzXML format using ProteoWizard software. Peak extraction was performed using XCMS software and extracted peaks were aligned to correct for minor differences in retention times due to variations in experimental conditions. Peak areas were corrected using the "Support Vector Regression" (SVR) method, and peaks with greater than 50% missing in each group of samples were filtered. Metabolite identification information is then obtained through various libraries such as self-built libraries and public databases as well as using metDNA methods.

### 2.4 Study design

The primary endpoint of this study is overall survival (OS), defined as the time from the start of the first treatment until the patients' death from any cause. The secondary endpoint was progression-free survival (PFS), defined as the time from the start of the first treatment to the patients' tumor progression (in any way) or death (in any way). We noticed that several papers mentioned 24-month survival rates and used this as a basis to assess the efficacy of lung cancer immunotherapy (20, 21), so combining the actual survival of 79 patients, we divided patients into Response (R = 41) and Non-Response (NR = 38) groups using 24-month cutoff values. Serum was collected from patients within four weeks prior to chemoimmunotherapy for untargeted metabolomics analysis. Combined with variable impact projection (VIP) and P values, differential metabolites were initially selected, and then the differential metabolites finally included in the model were further selected by LASSO and RF methods (22). Patients were randomly divided into training and validation sets at a ratio of 7:3, modeled using multiple machine learning methods such as RF, SVM, and LR, and then receiver operating characteristic (ROC) curves were plotted to assess model performance. The best model tested by different machine learning methods was selected, then the risk score was calculated, and the total population was divided into low-risk and high-risk groups using the median as the cutoff. Comparison of prognostic differences between high-risk and low-risk groups further assessed model performance.

### 2.5 Data analysis and statistical methods

Baseline characteristics of patients were compared using Chi-square test or Fisher 's exact test. OS and PFS were calculated using Kaplan-Meier method and log-rank test. Univariate and multivariate Cox regression were used to assess the impact of different factors on OS and PFS. ROC curves were utilized to evaluate the predictive capability of each factor. Specifically, the model's classification ability was demonstrated by plotting the relationship between the true positive rate (sensitivity) and the false positive rate (1-specificity). The area under the curve (AUC) was subsequently calculated, with a higher AUC value indicating a stronger predictive ability of the model. Specifically, we followed the procedure outlined below: we generated 1000 bootstrap samples from our dataset. For each bootstrap sample, we calculated the AUC. We sorted the AUC values obtained from the bootstrap samples and determined the 2.5^th^ and 97.5^th^ percentiles to obtain the 95% confidence interval (CI). Let AUC denote the area under the ROC curve, and let CI_lower_ and CI_upper_ denote the lower and upper bounds of the 95% CI, respectively. The results can be expressed as: 95% CI=[CI_lower_,CI_upper_].

Orthogonal partial least squares discriminant analysis (OPLS-DA) was used to demonstrate differences between groups, which was accomplished using the MetaboAnalystR package in R software. The volcano plot primarily serves to illustrate the relative content disparities of metabolites between two sets of samples, alongside the statistical significance of these differences. Student's t-test was employed to analyze variables with a normal distribution and equal variance between the two groups, whereas the Mann-Whitney test was utilized for those with a non-normal distribution. Significant differences in selecting differential metabolites were determined by VIP > 1 and p-value < 0.05. The annotation of metabolic pathways, encompassing the differential metabolites, was executed with the aid of the Kyoto Encyclopedia of Genes and Genomes (KEGG) database. LASSO regression was conducted using the glmnet, foreign, and tidyr packages in R, while the RF method was executed via the varSelRF package in R. The R package used by SVM is mainly e1071 package, and the R package used by LR is mainly glm package.

All data analyses were conducted utilizing R version 4.4.1 and SPSS version 26.0, and Adobe Illustrator 2022 was used for picture drawing integration. A p-value less than 0.05 in a two-tailed test was deemed statistically significant.

## 3 Results

### 3.1 Characteristics of the study set

**Figure 1:**
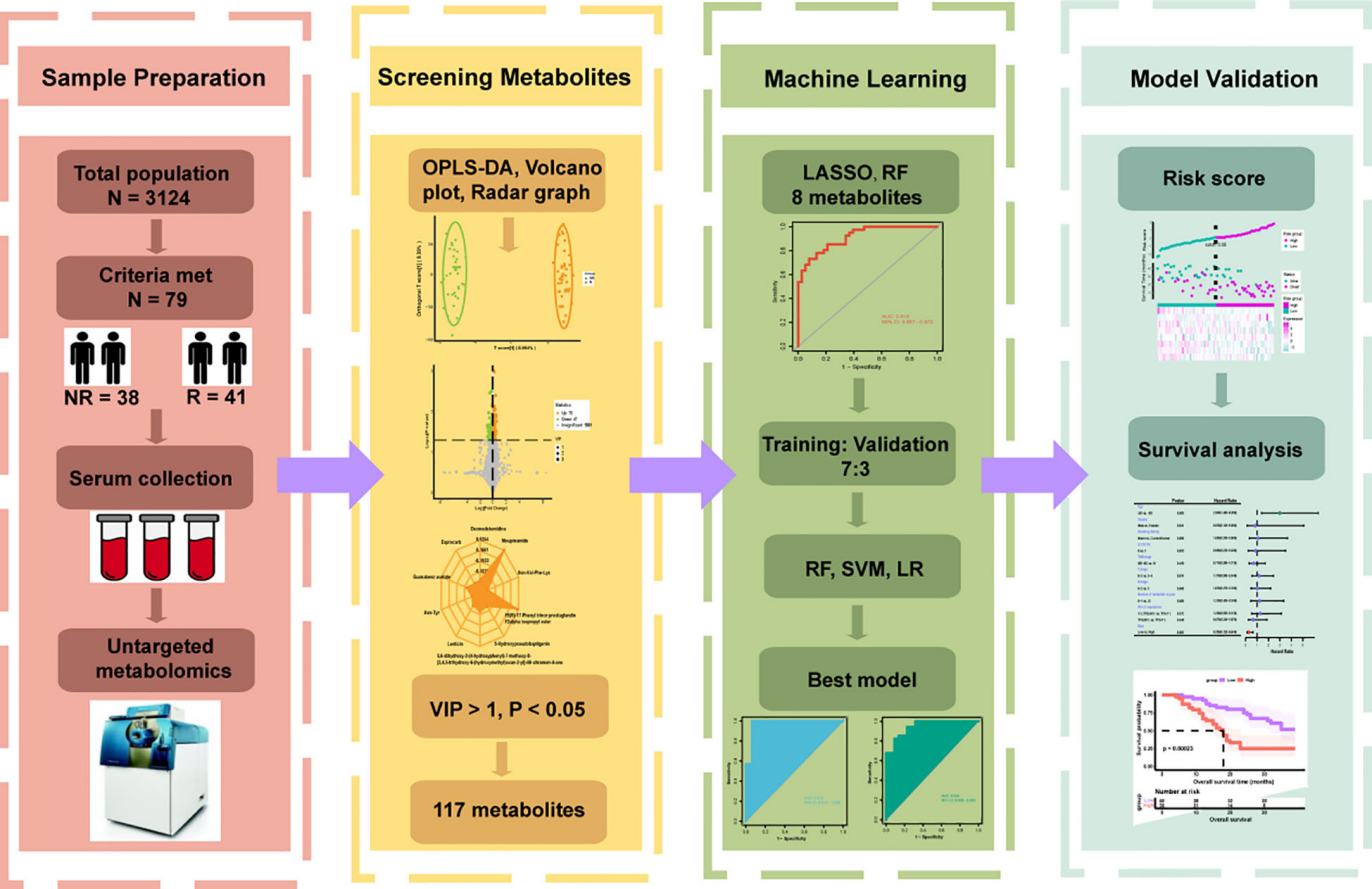
Flowchart of the whole study. According to the inclusion and exclusion criteria, 79 of 3124 patients were enrolled and divided into Response (R = 41) and Non-Response (NR = 38) groups based on overall survival. Serum was collected for untargeted metabolomics analysis before they underwent immunotherapy combined with chemotherapy. Orthogonal partial least squares discriminant analysis (OPLS-DA), volcano plot and radar plot could reflect the differences in metabolic characteristics between the two groups, and 117 differential metabolites were preliminarily selected according to variable importance in projection (VIP) > 1, P < 0.05. Various machine learning algorithms were used to construct the best prediction model and test the prediction performance in combination with clinical features.

The overall flow of our study was shown in **Figure 1**. There were 79 patients in our study who received combination therapy as their first line of treatment. Their baseline clinical characteristics such as age, sex, smoking history, ECOG PS score, TNM stage, number of metastatic organs and PL-L1 expression were shown in **Table 1**. As can be seen from the **Table 1**, our selected population was concentrated at ≥ 65 years, male, with a history of smoking, ECOG PS 0, TNM stage IV, and PD-L1 expression ≥ 1%. In **Table 2**, after we divided 79 patients into NR (OS < 24 months) and R (OS ≥ 24 months) groups according to OS, we then compared the clinical characteristics of the two groups and performed a chi-square test and found that these baseline characteristics were evenly distributed in both groups without statistical difference (P > 0.05).

**Table 1:** Baseline clinical characteristics.

Characteristic	Patients
Total number	79
Age (years), n (%)	
<65	27 (34.2)
≥65	52 (65.8)
Gender, n (%)	
Male	73 (92.4)
Female	6 (7.6)
Smoking history	
Never	15 (19.0)
Current/former	64 (81.0)
ECOG PS, n (%)	
0	72 (91.1)
1	7 (8.9)
TNM stage, n (%)	
IIIB-IIIC	25 (31.6)
IV	54 (68.4)
T stage, n (%)	
0-2	33 (41.8)
3-4	46 (58.2)
N stage, n (%)	
0-2	49 (62.0)
3	30 (38.0)
Number of metastatic organs, n (%)	
0-1	55 (69.6)
≥ 2	24 (30.4)
PD-L1 expression, n (%)	
TPS< 1%	17 (21.5)
1%≤ TPS≤ 49%	24 (30.4)
TPS≥ 50%	18 (22.8)
Unknown	20 (25.3)

ECOG PS, eastern cooperative oncology group performance status; PD-L1, programmed cell death-ligand 1; TPS, tumor proportion score.

**Table 2:** Comparison of clinical characteristics between non-response (NR) and response (R) groups.

Characteristic	NR group (n=38)	R group (n=41)	P
Age (years), n (%)			
< 65	14 (36.8)	13 (31.7)	0.631
≥ 65	24 (63.2)	28 (68.3)	
Gender, n (%)			
Male	34 (89.5)	39 (95.1)	0.420
Female	4 (10.5)	2 (4.9)	
Smoking history			
Never	8 (21.1)	7 (17.1)	0.652
Current/former	30 (78.9)	34 (82.9)	
ECOG PS, n (%)			
0	34 (89.5)	38 (92.7)	0.705
1	4 (10.5)	3 (7.3)	
TNM stage, n (%)			
IIIB-IIIC	13 (34.2)	12 (29.3)	0.637
IV	25 (65.8)	29 (70.7)	
T stage, n (%)			
0-2	18 (47.4)	15 (36.6)	0.332
3-4	20 (52.6)	26 (63.4)	
N stage, n (%)			
0-2	22 (57.9)	27 (65.9)	0.466
3	16 (42.1)	14 (34.1)	
Number of metastatic organs, n (%)			
0-1	30 (78.9)	25 (61.0)	0.083
≥ 2	8 (21.1)	16 (39.0)	
PD-L1 expression, n (%)			
TPS< 1%	7 (18.4)	10 (24.4)	0.896
1%≤ TPS≤ 49%	14 (36.8)	10 (24.4)	
TPS≥ 50%	7 (18.4)	11 (26.8)	
Unknown	10 (26.4)	10 (24.4)	

NR, non-response; R, response; ECOG PS, eastern cooperative oncology group performance status; PD-L1, programmed cell death-ligand 1; TPS, tumor proportion score.

### 3.2 Comparison of the serum metabolic profiles between NR and R groups

**Figure 2:**
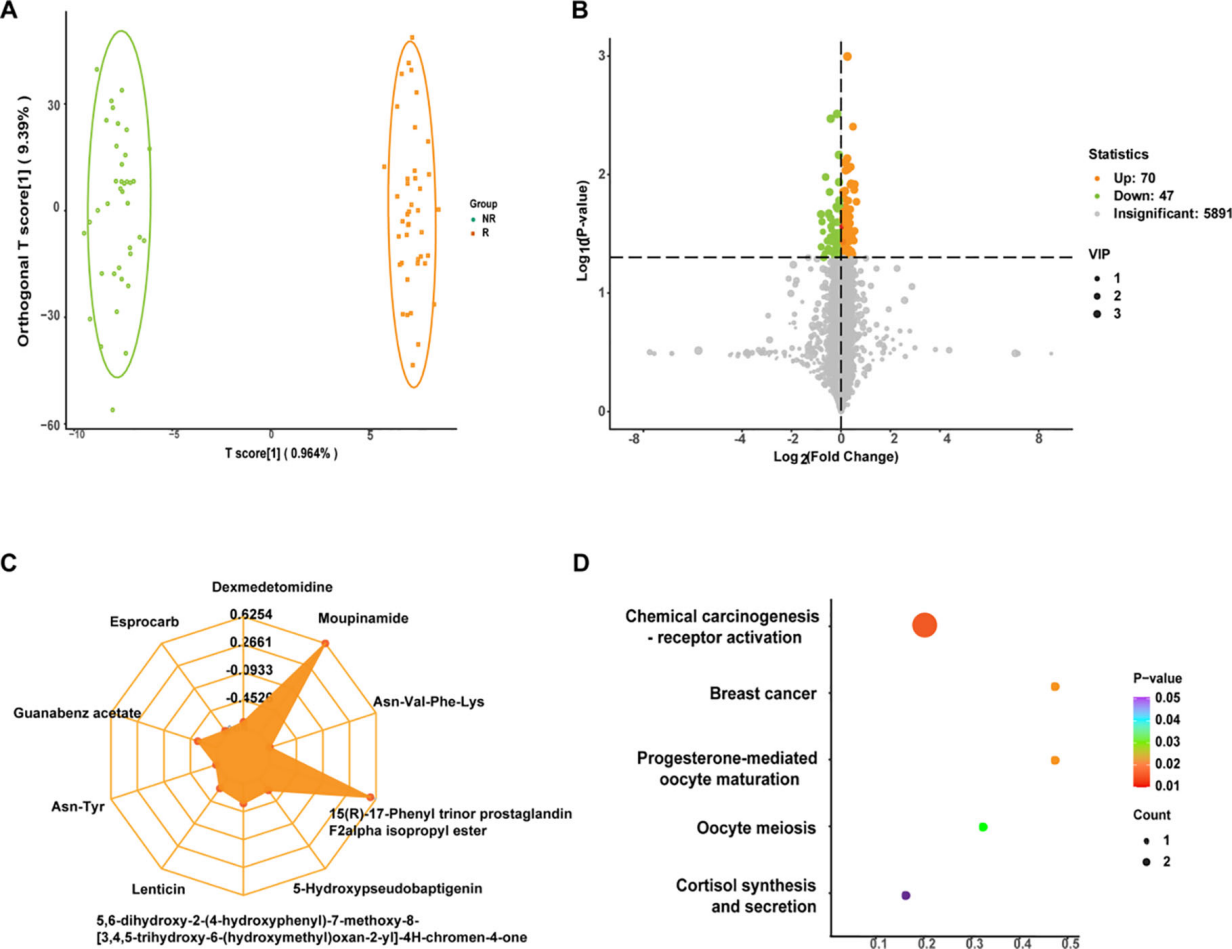
**(A)** Orthogonal partial least squares discriminant analysis (OPLS-DA) plots for non-response (NR) and response (R) groups. The abscissa represented the score of the predicted component, and the abscissa direction could see the gap between groups; the ordinate represented the score of the orthogonal component, and the ordinate direction could see the gap within the group; and the percentage represented the interpretation rate of the component to the dataset. **(B)** Volcano plot of differential metabolites. Each point represented a metabolite, where green, yellow, and gray points represented down-regulated, up-regulated, and metabolites that could be detected but were not significantly different, respectively; abscissa represented the log value of the fold difference in the relative content of a metabolite between the two groups of samples, ordinate indicated the level of significance of the difference, and the size of the dot represented the variable importance in projection (VIP) value. **(C)** Differential metabolite radar plot. Grid lines correspond to log_2_FC, that was, the fold difference of differential metabolites was logarithmic-ally valued at the base of 2, and yellow shading consisted of log_2_FC lines for each substance. **(D)** Differential metabolite Kyoto Encyclopedia of Genes and Genomes (KEGG) enrichment plot. The abscissa represented the Rich Factor corresponding to each pathway, the ordinate was the pathway name (sorted by P-value), and the color of the dots was the P-value size, with red indicating more significant enrichment. The size of the dots represented the number of differentially enriched metabolites.

In order to initially screen for differential metabolites between the two groups, we first verified the rationality of grouping, and the OPLS-DA model showed that the two groups could be significantly separated (**Figure 2A**), demonstrating that patients with different survival periods had different metabolic profiles. Based on the VIP obtained from the OPLS-DA model (biological replicates ≥ 3), combined with the P-value of univariate analysis (biological replicates ≥ 2), we could initially screen 117 differential metabolites between the two groups (VIP > 1 and P < 0.05). Among them, as shown in volcano plot (**Figure 2B**), 70 differential metabolites were up-regulated and 47 differential metabolites were down-regulated. In addition to performing statistics on VIP values and P-values for differential metabolites, we also calculated FC values for differential metabolites and plotted radar plots for the top 10 metabolites with the largest difference, i.e., the largest absolute log_2_FC value. In **Figure 2C**, these 10 metabolites were Moupinamide, 15(R)-17-Phenyl trinor prostaglandin F2alpha isopropyl ester, Guanabenz acetate, 5,6-dihydroxy-2-(4-hydroxyphenyl)-7-methoxy-8-[3,4,5-trihydroxy-6-(hydroxymethyl)oxan-2-yl]-4H-procen-4-one, 5-Hydroxypseudobaptigenin, Lenticin, Dexmedetamide, Esprocarb, Asn-Tyr, and Asn-Val-Phe-Lys. In addition, we also compared metabolic pathways, and KEGG metabolic pathways were significantly differentially enriched in metabolites between NR and R groups. The closer the P-value is to 0, the more significant the enrichment (**Figure 2D**). The first five pathways with the smallest size from small to large were Chemical carcinogenesis-receptor activation, Breast cancer, Progesterone-mediated oocyte maturation, Oocyte meiosis, and Cortisol synthesis and secretion.

### 3.3 Analysis of clinical factors affecting the efficacy of NR group and R group

**Figure 3:**
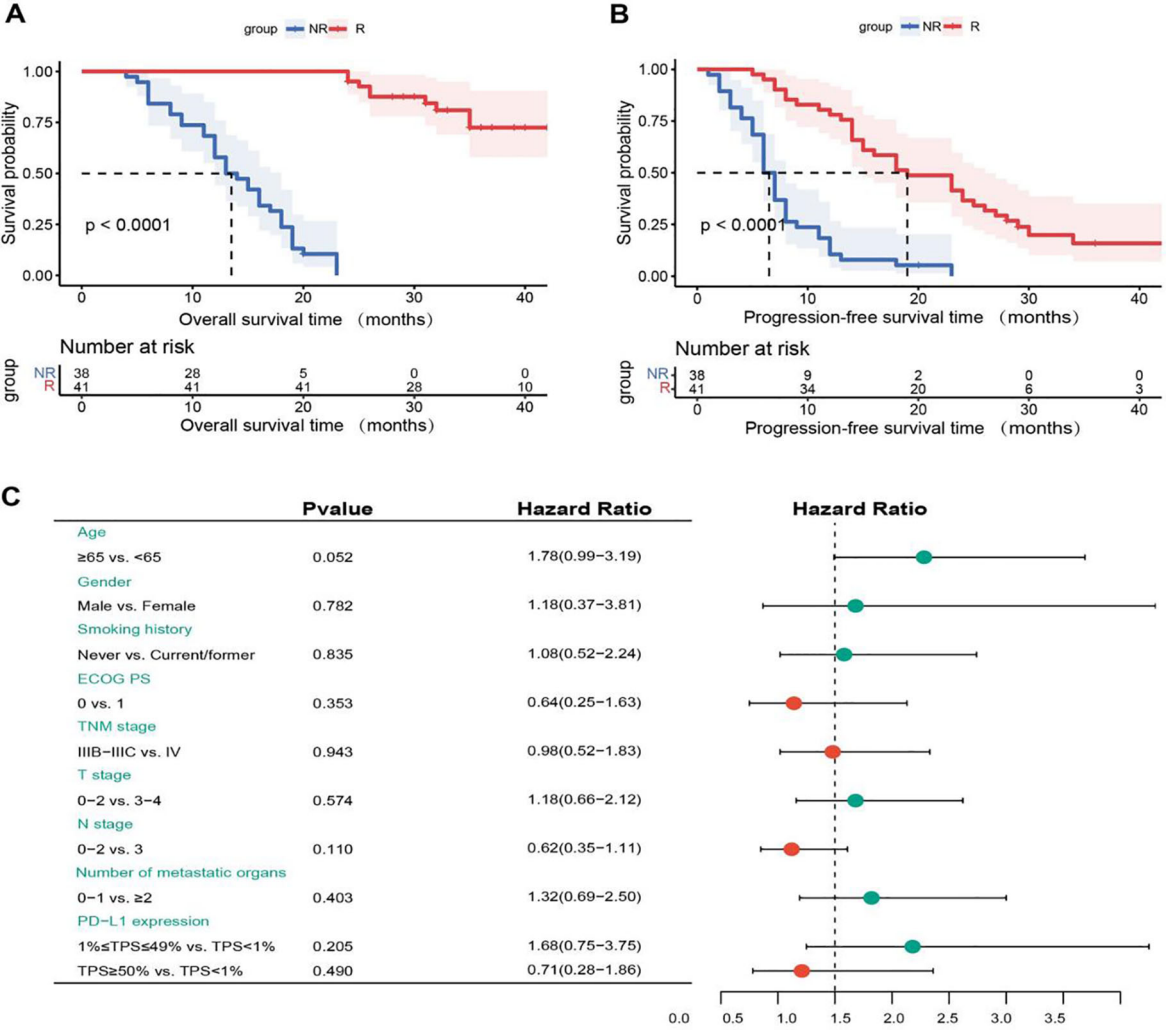
**(A)** Kaplan-Meier overall survival (OS) curves for non-response (NR) and response (R) groups. **(B)** Kaplan-Meier progression-free survival (PFS) curves for NR and R groups. **(C)** Univariate Cox regression analysis of OS. NR, non-response; R, response; ECOG PS, eastern cooperative oncology group performance status; PD-L1, programmed cell death-ligand 1; TPS, tumor proportion score.

In our study, the **Figure 3A** revealed that the median OS was 13 months (95% CI, 10.0-16.0) in the NR group, which was significantly shorter than that in the R group (P < 0.0001). In addition, median PFS was 6.0 months (95% CI, 5.0-7.0) versus 19.0 months (95% CI, 12.7-25.3) in NR group and R group, respectively (P < 0.0001). Therefore, OS was also significantly shorter in the NR group than in the R group and the data was shown in **Figure 3B**. To identify which clinical factors influence patient survival, we performed univariate Cox regression analysis and found no features to be significantly associated with survival (**Figure 3C**).

### 3.4 Building biomarker models based on machine learning

**Figure 4:**
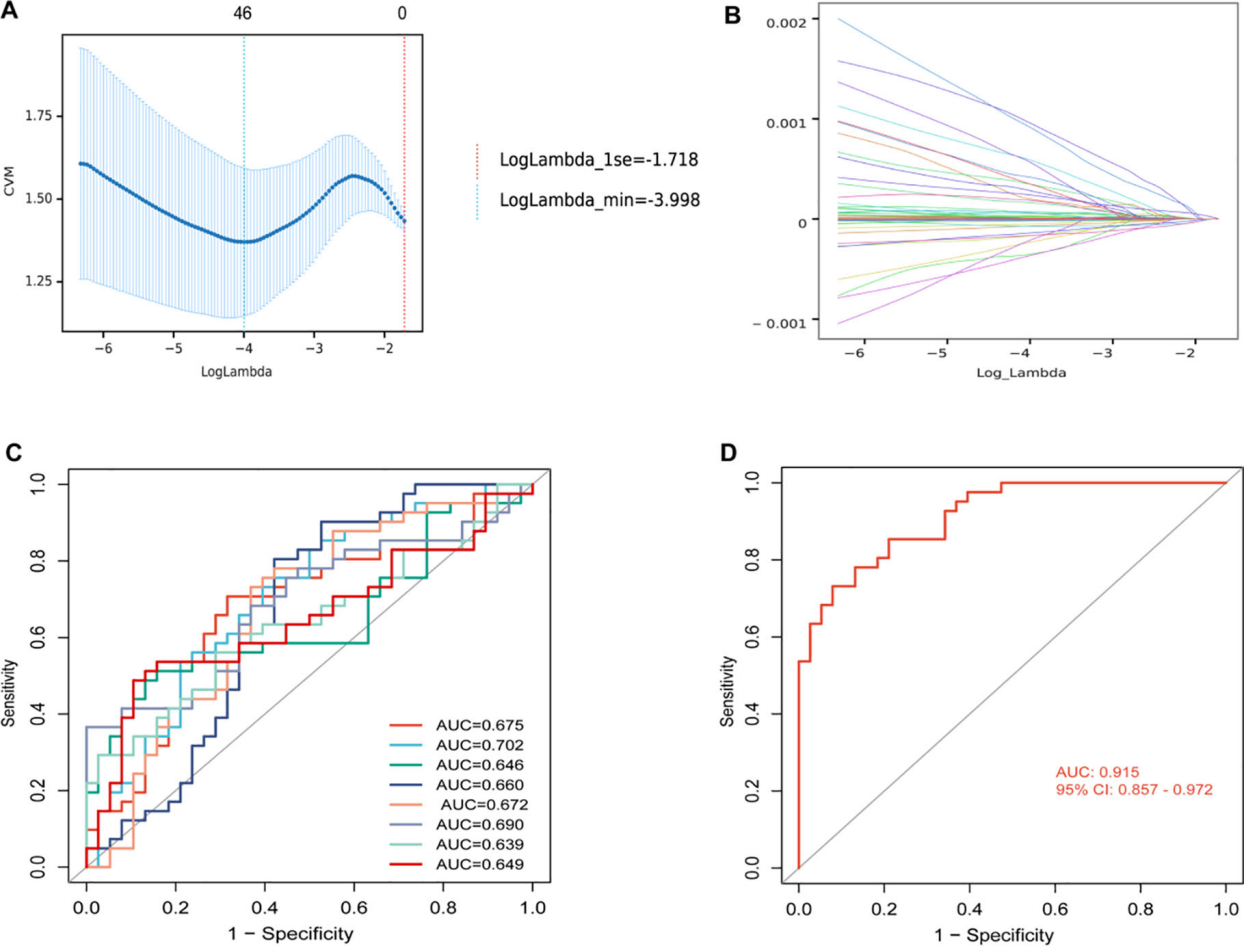
**(A)** Mean squared error plot for least absolute shrinkage and selection operator (LASSO) binomial regression lambda values. The horizontal axis was the logarithm of lambda, the vertical axis was the mean square error, and the two dashed lines were the maximum lambda value with the minimum lambda value and the mean error within one standard deviation, respectively. As the lambda value increased, the mean square error increased. **(B)** LASSO binomial regression lambda value coefficient plot. The horizontal axis was the log of lambda and the vertical axis was the coefficient of variation. As lambda increased, the variable coefficients decreased continuously and some variable coefficients changed to 0. **(C)** Receiver operating characteristic (ROC) analysis for each of 8 differential metabolites. **(D)** ROC analysis of models with 8 differential metabolite compositions. AUC, area under curve; CI, confidence interval.

Because the univariate Cox analysis was not significant, we did not include clinical characteristics in the model. Our preliminary statistical screening with p < 0.05, VIP > 1 resulted in a large number of metabolites, 117. To further screen more reliable biomarkers, we first selected 46 metabolites using LASSO (**Figures 4A, B**) and then identified metabolites using RF, and their cross-metabolites were candidate metabolites, and a total of 8 differential metabolites were identified as the best biomarkers. To avoid over-fitting and false positives, 500 feature selections and 10 cross-validations were performed. These 8 differential metabolites were Mevalonate, 1,2−Ethanedithiol, 4alpha−Hydroxymethyl−4beta−methyl−5alpha−cholesta−8,24−dien−3beta−ol, Arg−Asp−Leu−Tyr−Ser, Donhexocin, Glu−Cys−Ala, TG(10:0/14:0/a−15:0)[rac], D−3−Hydroxykynurenine, respectively, and their predictive performance was verified by AUC of ROC curves. As can be seen from **Figure 4C**, the predictive performance of individual differential metabolites is not sufficiently satisfactory (AUC < 0.75). However, the model formed by their combination had good predictive performance, with AUC values reaching 0.915, (95% CI, 0.857-0.972), suggesting that this model may be used as a predictive panel to predict the efficacy of immunotherapy combined with chemotherapy in patients with advanced lung squamous cell carcinoma (**Figure 4D**).

**Figure 5:**
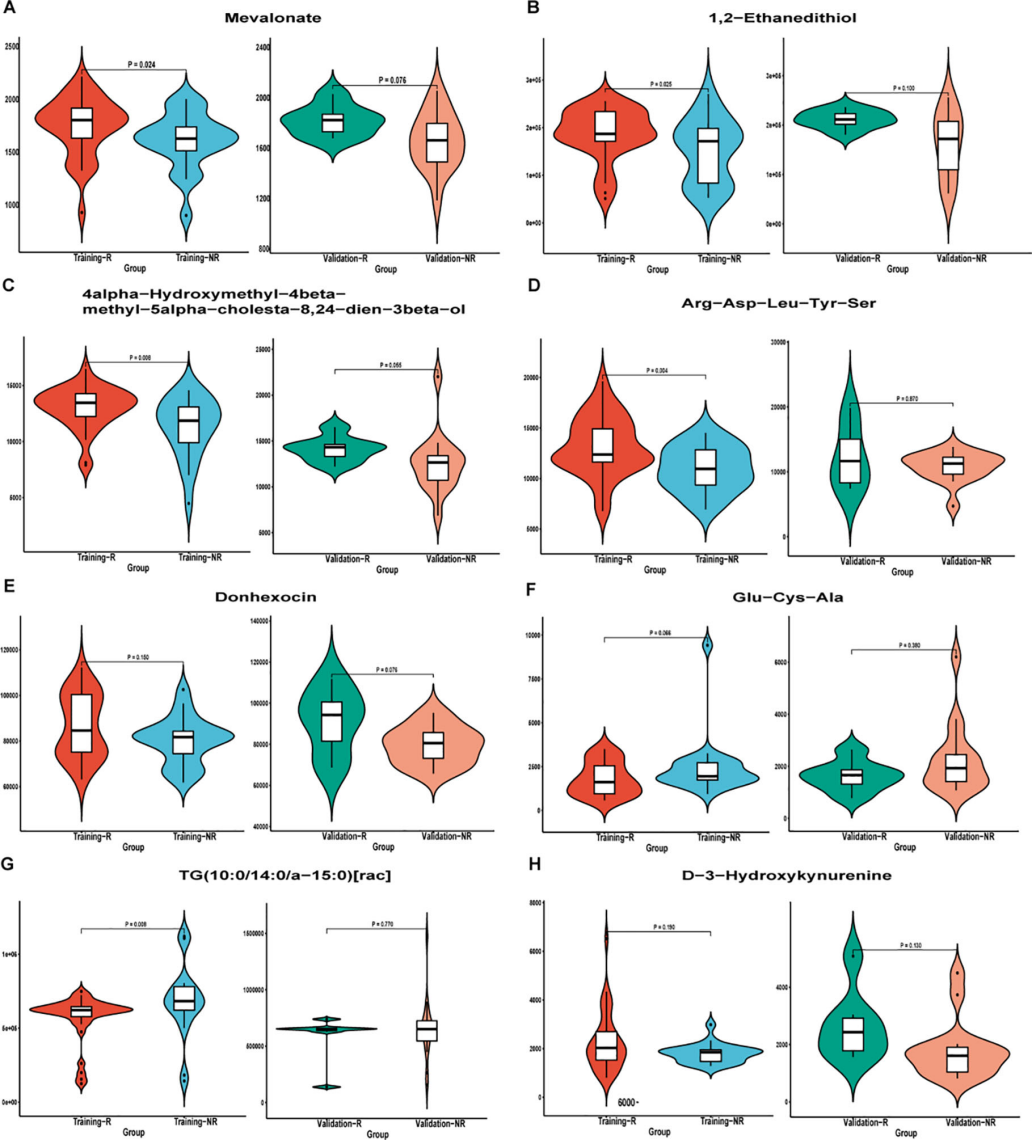
Differential metabolite violin plot of **(A)** Mevalonate; **(B)** 1,2−Ethanedithiol; **(C)** 4alpha−Hydroxymethyl−4beta−methyl−5alpha−cholesta−8,24−dien−3beta−ol; D) Arg−Asp−Leu−Tyr−Ser; **(E)** Donhexocin; **(F)** Glu−Cys−Ala; **(G)** TG(10:0/14:0/a−15:0)[rac]; **(H)** D−3−Hydroxykynurenine in the training and validation sets. Abscissa was sample grouping and ordinate was relative content of differential metabolites (original peak area). NR, non-response; R, response.

**Figure 6:**
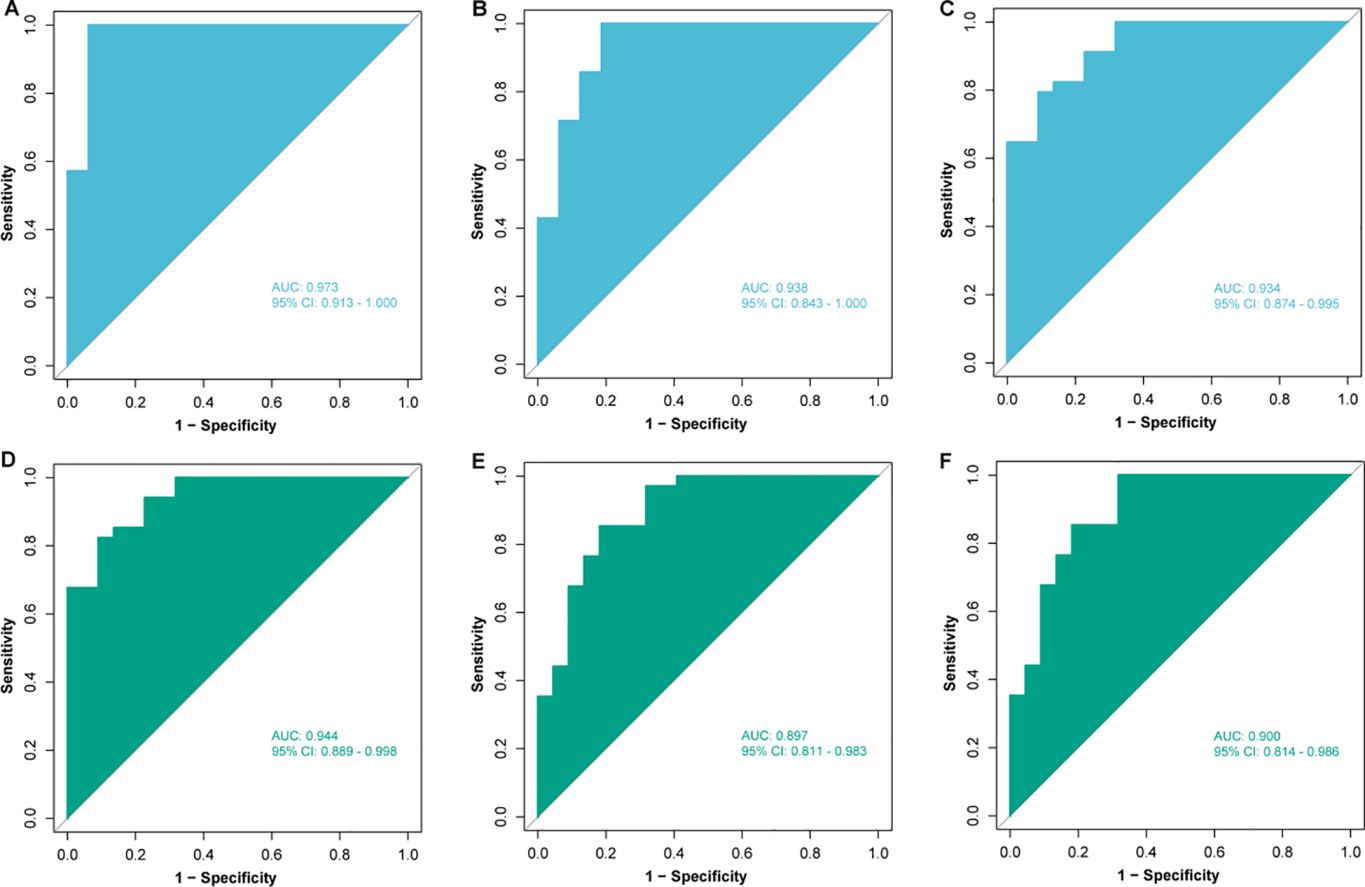
Construction and validation of models based on different machine learning algorithms. Receiver operating characteristic (ROC) analysis of **(A)** random forest (RF), **(B)** support vector machine (SVM) and **(C)** logistic regression (LR) models in the training set; ROC analysis of **(D)** RF, **(E)** SVM and **(F)** LR models in the validation set. AUC, area under curve; CI, confidence interval.

Subsequently, patients (n = 79) were randomly divided into training set (n = 55) and validation set (n = 24) in a 7:3 ratio, and various machine learning models were employed for datasets consisting of these eight metabolites, including RF model, SVM model, and LR model. The comparison of the relative content of these eight metabolites between the NR and R groups was generally consistent in the training and validation sets, but most of them were not significantly different (**Figure 5**), still suggesting that single metabolites were not able to be biomarkers. However, the RF, SVM, LR models constructed by the panel of eight metabolites showed good predictive performance, with AUCs of 0.973, 0.938, and 0.934 in the training set and 0.944, 0.897, and 0.900 in the validation set (**Figure 6**), with the RF model having the best predictive performance. The accuracy, precision, recall and F1 values of RF model in the training set were 0.98, and the accuracy, precision, recall and F1 values in the validation set were 0.96, 1.00, 0.96 and 0.98, respectively.

### 3.5 Identifying patient survival outcomes based on model

**Figure 7:**
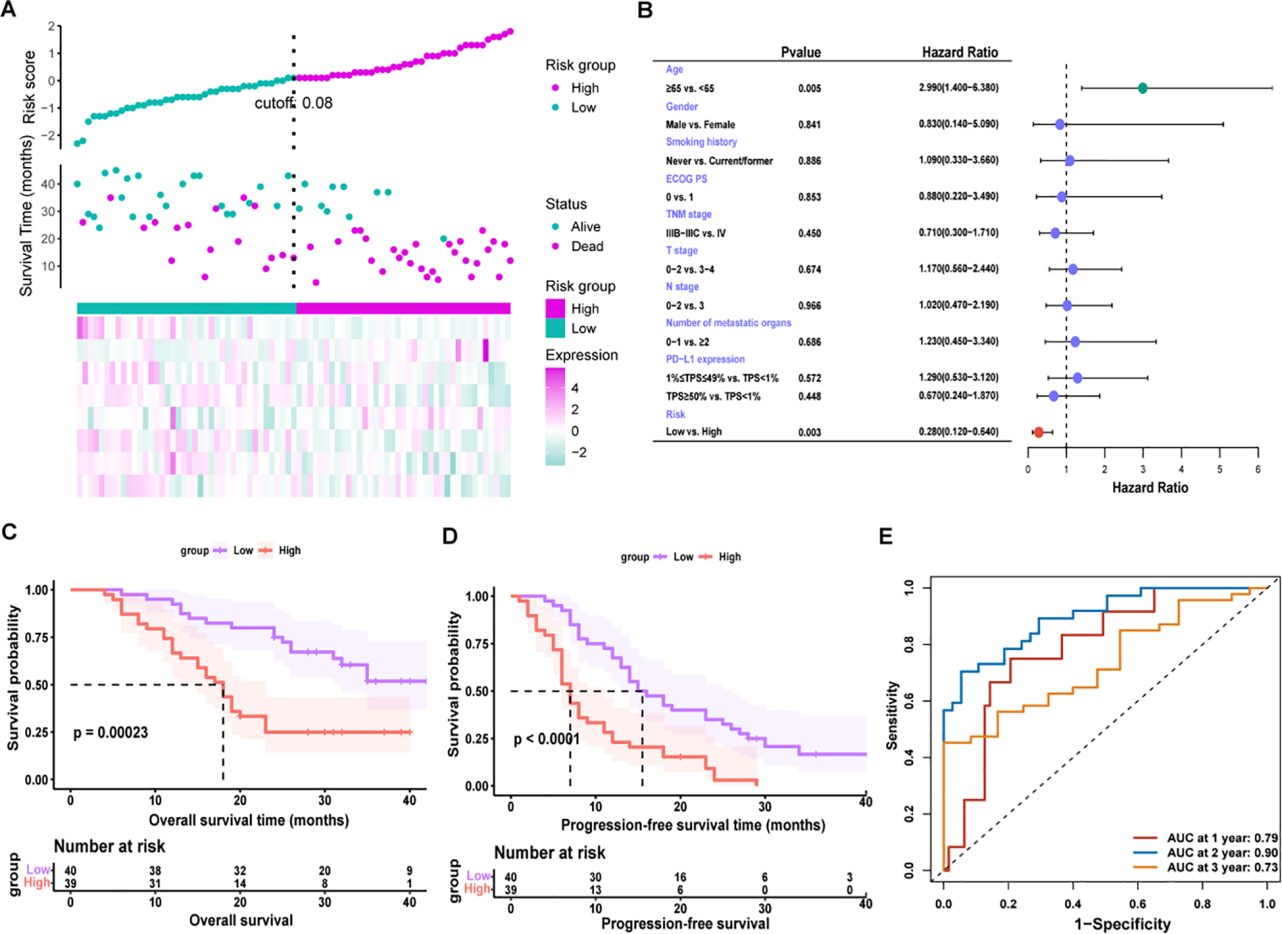
**(A)** Risk score triad plot. Patients were divided into low-risk (n = 40) and high-risk (n = 39) groups using the median of the risk score as the cutoff value. **(B)** Univariate Cox regression analysis of overall survival (OS). **(C)** Kaplan-Meier OS curves for low-risk and high-risk groups. **(D)** Kaplan-Meier progression-free survival (PFS) curves for low-risk and high-risk groups. Patients (n = 79) were divided into low-risk (n = 40) and high-risk (n = 39) groups according to the median risk score (cutoff = 0.08). **(E)** Time-dependent receiver operating characteristic (ROC) curve analysis for the prognostic value of the model for different years. NR, non-response; R, response; ECOG PS, eastern cooperative oncology group performance status; PD-L1, programmed cell death-ligand 1; TPS, tumor proportion score; AUC, area under the curve.

In **Figure 7A**, to further investigate the relationship between relative combined metabolite panel content and patient survival outcomes, we obtained risk scores by constructing a risk model and divided patients into low-risk (n = 40) and high-risk (n = 39) groups using the median of risk scores as the cutoff value. All baseline factors and risk factors were included in the multivariate Cox analysis (**Figure 7B**), and age > 65 years was significantly associated with shorter overall survival (HR, 2.990; 95% CI, 1.440-6.380; P = 0.005), whereas the low-risk group had significantly longer survival than the high-risk group (HR, 0.280; 95% CI, 0.120-0.640; P = 0.003). Multivariate Cox regression analysis indicated that risk score and age were independent prognostic factors for OS. **Figures 7C, D** present OS and PFS comparisons between the high-risk and low-risk groups. The median OS in the high-risk group was 18.0 months (95% CI, 15.0-21.0), which was significantly shorter than that observed in the low-risk group (P = 0.00023). In the high-risk group, median PFS was 7.0 months (95% CI, 5.9-8.1), which was significantly lower than PFS in the low-risk group, which was 15 months (95% CI, 10.0-20.0), and the difference was statistically significant (P < 0.0001). Time-dependent ROC curves indicated AUCs of 0.79, 0.90, and 0.73 at the 1-year, 2-year, and 3-year intervals, respectively, for the risk factors in forecasting OS (**Figure 6E**). These results indicated that our model could potentially predict the survival outcomes of patients.

## 4 Discussion

In this investigation, we employed untargeted metabolomics to explore metabolic changes in the serum of patients with advanced lung squamous cell carcinoma who received chemoimmunotherapy with varying degrees of effectiveness, aiming to identify biomarker models that could potentially serve as prognostic indicators. In particular, we used machine learning techniques to analyze serum metabolites, thereby enhancing the precision of our identified biomarkers. We developed a panel comprising 8 metabolites that demonstrated high accuracy in both the training and validation sets. Furthermore, the risk score derived from this model serves as an independent prognostic marker, capable of effectively differentiating patients with varying survival outcomes with remarkable reliability and precision.

Lung cancer stands as a primary contributor to cancer-related mortality in China and across the globe (23). Specifically, lung squamous cell carcinoma presents a huge challenge in treatment due to its complexity and heterogeneity (24). Moreover, lung squamous cell carcinoma exhibits fewer genetic mutations, which often renders it less responsive to targeted therapies compared to lung adenocarcinoma. Increasing studies have established the place of immunotherapy combined with chemotherapy in the first-line treatment of advanced lung squamous cell carcinoma (25, 26), and this combined approach has been shown to substantially enhance both patients’ survival rates and PFS. Consequently, it is imperative to identify biomarkers capable of predicting the efficacy of chemoimmunotherapy in patients with advanced lung squamous cell carcinoma with greater accuracy and convenience. Combining metabolomics and machine learning to construct biomarker models has already shown potential in the field of lung cancer, and many studies have made progress. In a recent study (27), the authors performed metabolomics and lipidomics studies on serum samples from 461 subjects (including NSCLC, SCLC, healthy participants) to establish a metabolomics/lipid-based diagnostic model. The machine learning algorithm was also used to validate the screening results, and the performance of candidate metabolites in the model was analyzed by ROC curves. The results showed that the AUC of RF model was 0.93, indicating that the model had good predictive ability. An another study employed supervised machine learning algorithms to construct classification models using metabolomics data (28). By contrasting the metabolomic profiles of patients with NSCLC against those of individuals without cancer, it was possible to pinpoint significant alterations in the concentration levels of metabolites involved in tryptophan metabolism, the tricarboxylic acid (TCA) cycle, the urea cycle, and lipid metabolism. Utilizing these identified metabolites and their respective proportions, a machine learning classification model with a remarkable ROC AUC value of 0.96 was successfully developed. These studies suggested that it was feasible to construct metabolomics and machine learning-based models to predict the efficacy of immune combination therapy in the field of lung squamous cell carcinoma, but we also found that previous studies had mainly been diagnostic models and had not been studied in predicting the direction of immunotherapy combined with chemotherapy in advanced lung squamous cell carcinoma.

Whereas our study considered the potential of metabolites as prognostic markers, it highlighted the predictive value of metabolites in immunotherapy combination therapy for advanced lung squamous cell carcinoma. Untargeted metabolomics analysis facilitated the measurement of thousands of metabolites, thereby enabling the identification of a multitude of potential biomarkers. Furthermore, we detected metabolites in peripheral blood, which was readily obtainable, non-invasive, and imposed less discomfort and risk on patients compared to invasive procedures requiring tissue biopsy. Peripheral blood biomarkers could reflect changes in the tumor and host microenvironment in real time and dynamically, allowing repeated, multiple sampling and enabling tracking of dynamic changes during treatment. Simultaneously, we integrated machine learning algorithms to analyze complex omics data, a process that proved to be swifter and more efficient than conventional manual methods, while also enhancing the stability and precision of our predictions (29, 30). In our study, we first compared the serum metabolites of patients with different efficacy using untargeted metabolomics, and it could be seen that the metabolites of the two groups were significantly different, and there were multiple differential metabolites and multiple differential metabolic pathways. However, multivariate analysis showed that clinical factors did not independently predict treatment outcome in these patients, so perhaps we could start with differential metabolites to find biomarkers for immunotherapy combination therapy.

To avoid overfitting, we first implemented the LASSO regression algorithm to select 46 out of 117 metabolites in total. Then, the RF algorithm was used to take their intersection, and the prediction panel containing 8 metabolites was preliminarily established. However, our results showed that the prediction performance of a single differential metabolite was not good, and only integrating these differential metabolites to build a model could better predict the efficacy. In particular, our study utilized three machine learning algorithms (RF, SVM and LR) to achieve the best combination of models. This approach enhanced the overall performance, leading to a more efficient and effective model. The RF model had high accuracy and generalization ability. By integrating multiple decision trees, it effectively reduced the risk of overfitting and enhanced the model’s generalization capability. It also demonstrated strong ability in processing high-dimensional data and resisting noise, and it could automatically assess the importance of features in prediction. However, it was computationally expensive, especially when dealing with large-scale datasets. Compared to LR, its prediction speed was slower. The SVM method showed excellent performance in high-dimensional space and was suitable for handling complex problems. It had strong generalization ability and could effectively deal with both nonlinear and linear separable data. However, it was characterized by high computational complexity and difficulty in parameter tuning. Additionally, it lacked interpretability compared to RF and LR. LR was simple, efficient, and interpretable. However, as a linear classifier, it might not achieve good fitting effects for data with complex nonlinear relationships between features and targets, and it was prone to underfitting. Taking into account the size of the dataset, prediction speed, and prediction performance in both the training and validation sets, we ultimately decided on the RF model.

Eventually, we developed a model containing 8 metabolites, including Mevalonate, 1,2-Ethanedithiol, 4alpha-Hydroxymethyl-4beta-methyl-5alpha-cholesta-8,24-dien-3beta-ol, Arg-Asp-Leu-Tyr-Ser, Donhexocin, Glu-Cy-Ala, TG(10:0/14:0/a-15:0) [rac], D-3-Hydroxykynurenine. Some of these substances were well-known, and others were unfamiliar metabolites that may be easily ignored or missed by traditional analytical methods. For example, the mevalonate pathway involved in mevalonate is one of the important pathways of cellular metabolism, which has been reported to regulate adaptive immunity, and confirmed that this pathway can be used as a new vaccine adjuvant and immunotherapy drug target (31). In addition, Donhexocin and D-3-Hydroxykynurenine primarily function in neurodegenerative diseases and are significant in the process of apoptosis (32, 33). In contrast, other metabolites have received scant research attention. We also discovered that eight metabolite biomarkers were poorly correlated, and none of the substances alone had a good predictive performance, which was related to the complexity of the tumor microenvironment (34), and a single biomarker was not sufficient to make a reliable prediction. Therefore, identifying a set of potential biomarkers would be more clinically meaningful for predicting the efficacy of immunotherapy, and kits could be developed based on this prediction model in the future and applied in practical clinical practice. Upon constructing the model, we conducted additional validation. We computed the risk score for each patient and employed the median risk score as a threshold to categorize them into high-risk or low-risk cohorts (35). Survival analysis demonstrated that patients in the high-risk group exhibited significantly shorter PFS and OS compared to those in the low-risk group, and Cox regression analysis confirmed that the risk score served as an independent predictor of outcome. In addition, this model also has good predictive performance for one-year, two-year and three-year survival rates of patients. Therefore, the excellent predictive value of this model was reconfirmed.

Certainly, there were some limitations to our study. First of all, this study was retrospective, and the sample size was small, being a single-center study, the results may have some bias, and a larger sample size multicenter prospective study should be conducted. Secondly, this study underwent internal validation only, with external validation being absent. It is possible that future efforts could further substantiate our findings through validation in an additional cohort or via basic experiments. Additionally, although our research indicated potential biomarkers for the combination of immunotherapy and chemotherapy. it was imperative that further functional studies be conducted to clarify metabolic mechanisms and to confirm the correlation between these metabolites and the progression of the disease.

## 5 Conclusions

To summarize, our study characterized the metabolic profile of patients with lung advanced squamous cell carcinoma using untargeted metabolomics and compared the accuracy of predictive models for immunotherapy combined with chemotherapy, constructed using three machine learning algorithms: RF, SVM, and LR. Among these models, the RF model achieved an AUC of 0.973 on the training set and 0.944 on the validation set, demonstrating the best prediction performance. Consequently, we used this method to construct a predictive model for the efficacy of immunotherapy combined with chemotherapy, including eight differential metabolites, which showed high accuracy in both the training and validation sets. Additionally, based on this model, we predicted survival outcomes for patients, revealing that survival was significantly longer in the low-risk group compared to the high-risk group (HR, 0.280; 95% CI, 0.120-0.640; P=0.003). Our study highlighted the potential value of leveraging machine learning-driven metabolomics to predict the effectiveness of chemoimmunotherapy for advanced lung squamous cell carcinoma, thereby offering a promising avenue for future clinical translation.

## Data Availability

The data presented in the study are deposited in the National Genomics Data Centre, CNCB repository (https://ngdc.cncb.ac.cn/), accession number OMIX009619.
